# A Clinical Comparative Study of Dexmedetomidine, Dexamethasone, and Clonidine as Adjuvants to Local Anesthetics in Supraclavicular Brachial Plexus Blocks

**DOI:** 10.7759/cureus.75718

**Published:** 2024-12-14

**Authors:** Umesh P Yadav, Jitendra Agrawal, Archana Singh, Preeti Goyal

**Affiliations:** 1 Anaesthesiology, Gajra Raja Medical College, Jaya Arogya Group of Hospitals, Gwalior, IND

**Keywords:** clonidine, dexamethasone, dexmedetomidine, ropivacaine, supraclavicular block

## Abstract

Introduction: The brachial plexus block is one of the peripheral blocks, beneath which the majority of upper limb surgical procedures are carried out. During upper limb surgery, a supraclavicular nerve block is an excellent substitute for general anesthesia.

Aim: This is a clinical comparative study of dexmedetomidine, dexamethasone, and clonidine as adjuvants to local anesthetics in supraclavicular brachial plexus block.

Material and methods: Ninety patients with American Society of Anesthesiologists (ASA) grades I and II scheduled for upper limb surgeries were randomly divided into three groups. Group RDEX (ropivacaine + dexmedetomidine; n = 30) received 20 ml of 0.5% inj. ropivacaine with 10 ml of 2% lignocaine and adrenaline with inj. dexmedetomidine 1 µg /kg (32 ml solution). Group RC (ropivacaine + clonidine; n = 30) received 20 ml of 0.5% inj. ropivacaine with 10 ml of 2% lignocaine and adrenaline with inj. clonidine 1 µg /kg (32 ml solution). Group RD (ropivacaine + dexamethasone; n = 30) received 20 ml of 0.5% inj. ropivacaine with 10 ml of 2% lignocaine and adrenaline with inj. dexamethasone 2 ml (8 mg) (32 ml solution).

Results: The onset of sensory and motor block was a statistically significant difference among the three groups (p < 0.001), showing that Group RDEX has the faster onset, followed by Group RC and Group RD as the slowest. There was a statistically significant difference in the mean duration of sensory and motor block among the three groups (p < 0.001), showing that Group RDEX has the longest average duration, followed by Group RC, while Group RD has the shortest. The comparison of the mean duration of analgesia shows a statistically highly significant difference among the three groups (p < 0.001). Dexmedetomidine (RDEX) has the longest duration of analgesia, followed by Group RC and then Group RD. The hemodynamic profile was comparable among the three groups. Sedation was observed in Groups RDEX and RC. Overall, no complications were found in Group RD.

Conclusion: Dexmedetomidine, when added to 0.5% ropivacaine for supraclavicular brachial plexus blocks, decreases the time of onset of sensory and motor blockade and prolongs the duration of the block and the duration of analgesia as compared to clonidine and dexamethasone with 0.5% ropivacaine, making it a better adjuvant to ropivacaine for upper limb surgeries.

## Introduction

The supraclavicular block is most suited for procedures in the arm and forearm. The brachial plexus is more dense in its trunks. As a result, a nerve block at this site can block more branches of the plexus resulting in rapid onset and complete block [1]. Ropivacaine, a newer amide local anesthetic with low lipid solubility, has gained popularity as it is less cardiotoxic and has a significantly higher threshold for central nervous system toxicity [2]. Many drugs have been used as adjuvants to increase the density, length, and speed of peripheral nerve blockade. Dexmedetomidine is an alpha-2 agonist. It is eight times more potent and selective than the partial alpha-2 agonist clonidine. When dexmedetomidine is used as an adjuvant to local anesthetic in regional blocks, the duration and postoperative analgesia are prolonged [3]. Clonidine is an imidazoline-alpha-2 receptor agonist. As an adjunct to local anesthetic in peripheral nerve block, it improves the efficacy and decreases the onset time [4]. Dexamethasone is a glucocorticoid with a long duration of action. It enhances the analgesic effect of local anesthetics [5]. The aims of this study were to compare the onset and duration of motor and sensory blockade and evaluate the efficacy of dexmedetomidine, dexamethasone, and clonidine in prolonging postoperative analgesia.

## Materials and methods

This double-blinded comparative study was done after obtaining written informed consent and getting approval from the Institutional Ethical Committee of Gajra Raja Medical College, Gwalior, Madhya Pradesh (certificate no. 117/IEC-GRMC/2022). The study was registered with the Clinical Trial Registry-India (CTRI/2024/01/062007). The study was performed on 90 patients of American Society of Anesthesiologists (ASA) grades I and II, aged 18 to 60 years, who were scheduled for upper limb surgeries. The study period was from September 2022 to July 2024. The sample size was 90, which was calculated using the following formula:

n = 2s²(Zα/2 + Z1 - β)² / μ1 - μ2,

where μ1 ​= mean duration of the sensory block among DX with ropivacaine = 10.4, μ2 = mean duration of the sensory block among DM with ropivacaine =12.78, Z α /2 = 1.96 (at 5% level of significance), Z1 - β = 1.28 (at 90% power of test), and S^2^ = 2.02

The procedure was explained to patients, and written consent was obtained. Patients were divided into three groups of 30 each by the closed-envelope method. Group RDEX (ropivacaine + dexmedetomidine; n = 30) received 20 ml of 0.5% inj. ropivacaine with 10 ml of 2% lignocaine and adrenaline with Inj. dexmedetomidine 1 µg /kg (32 ml solution), Group RC (ropivacaine + clonidine; n = 30) received 20 ml of 0.5% inj. ropivacaine with 10 ml of 2% lignocaine and adrenaline with inj. clonidine 1 µg /kg (32 ml solution), and Group RD (ropivacaine + dexamethasone; n = 30) received 20 ml of 0.5% inj. ropivacaine with 10 ml of 2% lignocaine and adrenaline with inj. dexamethasone 2 ml (8 mg) (32 ml solution).

The inclusion criteria for the study consisted of 90 patients aged 18-60 years, classified as ASA grade I or II. Patients were excluded from the study if they met any of the following conditions: refused to provide consent, had a history of allergic reaction to local anesthetic, had a history of psychiatric illness, or suffered from renal or liver impairment or cardiac disease. In addition, patients with coagulation abnormalities were excluded from participation. A pre-anesthetic assessment of the patients was performed. In the operating room, an 18G IV cannula was inserted, and intravenous fluid was connected. Standard monitoring was done throughout the procedure. The patients were placed in the supine position. All aseptic precautions were taken. Two percent lidocaine 2 ml was infiltrated. A nerve stimulator connected to 22 G, a 50-mm-long needle, was inserted 2 cm above the midclavicular point. It was directed lateral to the subclavian artery, caudally and medially. A muscle twitch response was elicited, and the study drug was injected after the blood was confirmed to have a negative aspiration. Following the injection of the drug intraoperatively and postoperatively, the following characteristics and outcomes were recorded.

The primary outcomes of the study included the onset and duration of the sensory block, the onset and duration of the motor block, the Visual Analogue Score (VAS score) for pain assessment, the sedation score, and any complications observed during the study. The secondary outcomes focused on monitoring and evaluating the hemodynamic parameters of the patients. Postoperative pain assessment was done with a VAS score of 0-10 where 0 = no pain, 1-3 = mild pain, 4-6 = moderate to severe pain, 7-9 = very severe pain, and 10 = worst pain possible [6]. The score was recorded in the postoperative period up to 12 hours. The sedation was assessed with the Ramsay sedation scale of 1-5 in which 1 = anxious and agitated/restless; 2 = cooperative, oriented, and tranquil; 3 = responsive to verbal commands and drowsy; 4 = asleep, responsive to light stimulation (loud noise, tapping); and 5 = asleep, slow response to stimulation, and no response to stimulation [1].

## Results

The results showed that the demographic data among the study groups were comparable with respect to age in years, sex, weight, ASA grade, and duration of surgery, as demonstrated in Table 1.

**Table 1:** Demographic profile of the patients in the three groups NS = nonsignificant, Group RDEX = ropivacaine + dexmedetomidine, Group RC = ropivacaine + clonidine, Group RD = ropivacaine + dexamethasone

Parameters	Group RDEX	Group RC	Group RD	P-value
Age (years)	38.07 ± 12.03	38.30 ± 12.18	34.20 ± 11.30	0.346 NS
Weight (kg)	58.87 ± 7.77	59.33 ± 6.88	59.03 ± 11.39	0.88 NS
Sex				0.38 NS
Male	18 (60%)	20 (66.67%)	23 (76.67%)	
Female	12 (40.0%)	10 (33.33%)	7 (23.33%)	
ASA grade				0.200 NS
Grade I	14 (46.7%)	14 (46.7%)	20 (66.7%)	
Grade II	16 (53.3%)	16 (53.3%)	10 (33.3%)	
Duration of surgery (min)	113.03 ± 33.24	97.00 ± 35.83	107.33 ± 16.80	0.115 NS

The results showed that the time of onset of sensory blockade was found to be rapid in Group RDEX at 3.53 ± 0.70 min, followed by Group RC at 7.17 ± 1.05 min and then Group RD at 8.50 ± 1.18 min (p < 0.05). The time of onset of motor blockade was found to be rapid in Group RDEX at 6.43 ± 1.04 min, followed by Group RC at 10.13 ± 1.46 min and then Group RD at 14.20 ± 1.17 min (p < 0.05). The duration of the sensory block was found to be prolonged in Group RDEX at 572.13 ± 30.94 min, followed by Group RC at 401.33 ± 17.51 min and then Group RD at 372.67 ± 21.68 min (p < 0.05). The duration of motor blockade was found to be prolonged in Group RDEX at 635.50 ± 30.125 min, followed by Group RC at 438.67 ± 53.109 min and then Group RD at 411.67 ± 22.335 min (p < 0.05). The duration of analgesia was found to be prolonged in Group RDEX at 647.67 ± 36.29 min, followed by Group RC at 456.00 ± 20.94 min and then Group RD at 410.33 ± 29.76 min (p < 0.05) (Table 2).

**Table 2:** Comparison of block characteristics among the three groups HS = highly significant, S = significant, Group RDEX = ropivacaine + dexmedetomidine, Group RC = ropivacaine + clonidine, Group RD = ropivacaine + dexamethasone

	Group	No.	Mean ± SD	F-value	P-value	Post-hoc Tukey test		
						RDEX to RC	RDEX to RD	RC to RD
Sensory onset	RDEX	30	3.53 ± 0.70	191.93	<0.001 HS	<0.001 HS	<0.001 HS	<0.001 HS
	RC	30	7.17 ± 1.05					
	RD	30	8.50 ± 1.18					
Sensory duration	RDEX	30	572.13 ± 30.94	603.563	<0.001 HS	<0.001 HS	<0.001 HS	<0.001 HS
	RC	30	401.33 ± 17.51					
	RD	30	372.67 ± 21.68					
Motor onset	RDEX	30	6.43 ± 1.04	253.087	<0.001 HS	<0.001 HS	<0.001 HS	<0.001 HS
	RC	30	10.13 ± 1.46					
	RD	30	14.20 ± 1.17					
Motor duration	RDEX	30	635.50 ± 30.125	317.868	<0.001 HS	<0.001 HS	<0.001 HS	0.018 S
	RC	30	438.67 ± 53.109					
	RD	30	411.67 ± 22.335					
Duration of analgesia	RDEX	30	647.67 ± 36.29	540.312	<0.0 01 HS	<0.001 HS	<0.001 HS	<0.001 HS
	RC	30	456.00 ± 20.94					
	RD	30	410.33 ± 29.76					

## Discussion

Peripheral nerve blocks have been found to be more effective with the addition of various adjuvants. Dexmedetomidine and clonidine are alpha-2 adrenoceptor agonists. However, dexmedetomidine is eight times more selective than clonidine. It has sedative, analgesic, antihypertensive, and antiemetic actions, and when used as an adjuvant, it decreases the anesthetic dose requirement. The possible mechanism of action was explained in a study conducted by Natarajan et al. [7] who discovered that clonidine acts as an analgesic and vasoconstrictor (via alpha-2 adrenergic receptors), reduces inflammation, and acts directly on the peripheral nerves.

Dexmedetomidine acts by hyperpolarizing the nerves by modifying transmembrane potential and ion conduction at locus ceruleus in the brain stem. Dexmedetomidine was found to be a better pharmacological agent due to its stable hemodynamic and enhanced sympathoadrenal stability, which decreases oxygen demand. Another agent that can be used as an adjuvant to increase the duration of local anesthetic analgesia is dexamethasone. Its action is mediated via glucocorticoid receptors. Soni et al. [8] reported that dexamethasone acts by decreasing transmission in unmyelinated C-fibers; it reduces ectopic neuronal activity and modifies potassium channel function in excitable cells. The demographic characteristics such as age, sex, weight, and ASA grade were comparable in our study in all the groups.

The mean onset of the sensory block and motor block showed a statistically significant difference among the three groups (p < 0.001), showing that Group RDEX has the faster onset of sensory and motor block while Group RD has the slowest onset. Our findings are in accordance with the study of R. Madhusudhan et al. [9], in which they found that the onset of sensory and motor blockade was faster in Group D, who received dexmedetomidine, and Group C, who received clonidine, which was statistically significant. Prapurna et al. [10] also found that the onset of sensory and motor block was faster with dexmedetomidine than with dexamethasone, which is in accordance with our study. Another study conducted by Agarwal et al. [2] found a faster onset of sensory and motor block in the ropivacaine-clonidine group than in the ropivacaine-dexamethasone group; this was in accordance with our study.

There was a statistically significant difference in the mean sensory and motor block duration among the study groups (p < 0.001), showing that Group RDEX has the longest average duration while Group RD has the shortest. Our result was in accordance with the study conducted by Jha et al. [11], Their study found that the mean duration of sensory and motor blockade was significantly prolonged in the bupivacaine-clonidine group compared to the bupivacaine-dexamethasone group. Another study by Hamada et al. [12] found that the mean duration of sensory block and motor block was prolonged in the dexmedetomidine group compared to the dexamethasone group. This result coincides with the result of our study. Shastri et al.'s [13] results were also in accordance with our study, which found that dexmedetomidine has a prolonged duration of the sensory and motor block compared to clonidine.

The mean duration of the analgesia showed a statistically highly significant difference in the mean among the study groups (p < 0.001). Dexmedetomidine (RDEX) has the longest, and RC has a longer duration than RD. In a study conducted by Mokkarala et al. [3], the mean duration of analgesia was significantly longer in the dexmedetomidine (DM) group than in the dexamethasone group (DS). This result coincides with our study. A similar result was found in another study by Kanvee et al. [14], in which the duration of postoperative analgesia was found to be longer in the dexmedetomidine group as compared to the group where patients received clonidine.

In our study, the hemodynamic profile was comparable among the three groups. The hemodynamic profile was also comparable in the studies conducted by Praneetha et al. [15] and Soni et al. [8] The mean Ramsay sedation score comparison among the three groups was statistically not significant (p > 0.05). A post-hoc Tukey test shows that dexmedetomidine (RDEX) as an adjuvant to local anesthetics resulted in higher sedation scores compared to dexamethasone (RD) at 20 and 30 minutes but not when compared to clonidine (RC). The result was similar to a study conducted by Hamada et al. [12] comparing dexmedetomidine and dexamethasone. In their study, they found that sedation scores were higher in patients receiving dexmedetomidine compared to the dexamethasone group. Prapurna et al. [10] found that all the patients in the dexmedetomidine group had sedation when compared to dexamethasone, which was statistically significant. This was in accordance with our study. In another study conducted by Rajoli et al. [1], a statistically significant difference was found in sedation scores between clonidine and dexamethasone. The sedation score was found to be higher in the clonidine group as compared to the dexamethasone group. This result coincides with the findings of our study. The mean heart rate, mean systolic blood pressure (SBP), mean diastolic blood pressure (DBP), mean arterial pressure (MAP), SPO_2_, and respiratory rate (RR) postoperative comparison among the three groups was statistically insignificant (p > 0.05), showing a comparable post-op hemodynamic parameter. The post-op hemodynamic profile was also comparable to the study conducted by Kanvee et al. [14]

Limitations of the study

This study did not involve the use of ultrasound guidance for administering the supraclavicular block. In addition, the patients' height was not taken into consideration during the procedure or analysis.

## Conclusions

From the present study, it was concluded that in supraclavicular brachial plexus blocks, dexmedetomidine has the fastest onset of sensory and motor blockage, followed by clonidine and then dexamethasone as adjuvants to 0.5% ropivacaine. Dexmedetomidine, when used as an adjuvant to 0.5% ropivacaine for supraclavicular brachial plexus block, increases the duration of sensory and motor blockage and the duration of postoperative analgesia, followed by clonidine and then dexamethasone. There were no hemodynamic changes among the groups. Sedation was observed in the dexmedetomidine and clonidine groups. No complications were observed in the dexamethasone group.
